# Prediction of basal metabolic rate in overweight/obese and non-obese subjects and its relation to pulmonary function tests

**DOI:** 10.1186/s13104-015-1320-8

**Published:** 2015-08-15

**Authors:** Tarig H Merghani, Azza O Alawad, Rihab M Ibrahim, Asim M Abdelmoniem

**Affiliations:** Department of Physiology, Faculty of Medicine, University of Tabuk, Tabuk, Saudi Arabia; Department of Pathology, Faculty of Medicine, University of Tabuk, Tabuk, Saudi Arabia; Department of Surgery, Faculty of Medicine, University of Tabuk, Tabuk, Saudi Arabia

**Keywords:** Basal metabolic rate, Lung function tests, Obese

## Abstract

**Background:**

Few studies investigated the association between basal metabolic rate (BMR) and indicators of pulmonary function. This study was conducted to estimate BMR in overweight/obese and non-obese healthy subjects using four commonly used predictive equations and to investigate its relation to the indicators of lung function tests (LFT). A cross sectional study was conducted in Tabuk University, Tabuk, Saudi Arabia. A total of 201 students (98 males and 103 females) participated in the study. Four different values of BMR were calculated for each participant using four different predictive equations (Harris-Benedict, Mifflin, FAO/WHO/UNU and Henry-Rees). A portable All-flow spirometer (Clement Clarke International, Harlow, UK) was used for measurements of LFT.

**Results:**

Significantly higher values of spirometric indicators (*p* < 0.05) were found in males compared to females, except for FEF75 and FEF75-85. Mean BMR values predicted with the four equations were significantly higher in the males compared to the females and among the overweight/obese compared to the non-obese subjects (*p* < 0.05). The relation between mean BMR values and the indicators of LFT was statistically insignificant (*p* > 0.05).

**Conclusion:**

Mean values of LFT indicators are not related to the estimated values of BMR. A practical calculation of BMR based on direct measurement of oxygen consumption is recommended to confirm the absence of this association.

## Background

The basal metabolic rate (BMR) is the main component of daily energy expenditure, accounting for 60–70 % of total energy expenditure in most individuals. Its measurement is essential in nutritional assessment and weight management programs. Accurate measurement requires strict laboratory preparations and special equipment. For this reason, predictive equations are increasingly used for its estimation in clinical practice. Historically, several predictive equations, based on body weight, height, and age were used for its estimation. These include equations created by Harris and Benedict [1], Mifflin [2], Food and Agriculture Organization/World Health Organization/United Nations University (FAO/WHO/UNU) [3], and Henry and Rees [4]. These equations are commonly used in clinical practice; however, they may overestimate the basal metabolic rate [5]. Mifflin equation was found to be the most accurate among all these equations [6].

Obesity is a leading preventable cause of mortality. Its prevalence is increasing worldwide. The estimated prevalence in Saudi Arabia is about 35.5 % [7]. Because of the direct influence of obesity on rate of metabolism, measurement of energy expenditure in obese subjects is an important step for estimation of their energy needs and weight control interventions. On the other hand, obesity and weight gain are associated with alterations in pulmonary function. They cause reduction in forced vital capacity (FVC) and forced expiratory volume in the first second (FEV1), changes in mechanics of respiration, decreased chest wall compliance, impaired exercise tolerance, and increased bronchial hyper-responsiveness [8, 9]. Recent studies suggest a strong association between obesity and asthma [10].

In spite of the wide applicability of metabolic rate measurements in clinical practice, there is limited information about metabolic rate estimations in overweight/obese and non-obese subjects in Saudi Arabia. On the other hand, there is a paucity of data regarding the association between basal metabolic rate values and indicators of pulmonary function. This study was conducted to estimate resting metabolic rate among overweight/obese and non-obese healthy subjects using four commonly used predictive equations and to investigate its relation to pulmonary function indicators.

## Methods

A cross sectional study was conducted in Tabuk University in Tabuk, Saudi Arabia. A total number of 201 students (98 males and 103 females) participated in the study. Inclusion criteria were healthy student, and age 18–23 years old. Exclusion criteria were age <18 or >23 years old, presence of skeletal deformity, current history of acute respiratory illness, and a history of a chronic disease.

The height and weight of each student were measured using standardized height and weight scales. The body mass index (BMI) was calculated for each student as weight (in kilograms) divided by square of height (in meters) [11]. The surface area was calculated using DuBios formula [12]. BMI less than 25.00 was classified as “non-obese” and 25.00 or higher as “overweight/obese”. A portable All-flow spirometer (Clement Clarke International, Harlow, UK) was used for measurement of FVC, FEV1, FEV1/FVC ratio, FEF and PEF for each subject. Spirometry measurements were carried out according to the guidelines of the American Thoracic Society (ATS) [13]. Four predictive equations were used for estimation of basal metabolic rate for all participants in the study as follows:

1. Harris and Benedict equation [1]: BMR (kcal/day) for men = (13.7516 wt/1 kg + 5.0033 ht/1 cm − 6.7550 age/1 year + 66.4730) and for women = (9.5634 wt/1 kg + 1.8496 ht/1 cm − 4.6756 age/1 year + 655.0955).
2. Mifflin equation [2]: BMR (kcal/day) = 10.0 wt/1 kg + 6.25 ht/1 cm − 5.0 age/1 year + s; (where s is +5 for males and −161 for females).
3. FAO/WHO/UNU equation [3]: BMR (kcal/day) for men (18–30 years old) = (0.0640 wt + 2.84) × 238.85, and for women (18–38 years old) = (0.0615 wt + 2.08) × 238.85.
4. Henry and Rees equation [4]: BMR (kcal/day) for men (18–30 years old) = (0.0560 wt + 2.800) × 238.85, and for women (18–38 years old) = (0.0480 wt + 2.562) × 238.85.

Data was analyzed with the Statistical Package for the Social Sciences, version 20 (SPSS Inc., Chicago, IL, USA). The Student’s t test was used for testing the statistical difference between the groups of males and females, and between the groups of “overweight/obese” and “non-obese” in relation to the demographic details, the spirometric indicators, and the predicted values of BMR for each equation. Statistical significance was accepted for p < 0.05. The research conforms to the ethical principles of medical research developed by the World Medical Association Declaration of Helsinki [14]. The research was approved and ethically cleared by the Deanship of Scientific Research and the Research Ethics Committee of Tabuk University/Saudi Arabia (reference number 003/2014). Written consents were obtained from the participants before entry into the study.

## Results

Table 1 shows characteristics of males and females in the study group. Males were significantly taller (*p* < 0.05) and heavier (*p* < 0.05) than females. Lung function test indicators were significantly higher (*p* < 0.05) among males compared to females, except for FEF75 and FEF75-85. Mean BMR values predicted with the four equations were significantly higher in males compared to females (*p* < 0.05). Table 2 shows insignificant statistical difference in age, height and lung function indicators (but not in weight and BMI) between overweight/obese and non-obese males and females of the study population. Mean BMR values for overweight/obese and non-obese subjects were presented in Table 3. A significant statistical difference was found between the two groups for each equation (*p* < 0.05). Tables 4 and 5 show the relation between mean BMR values and LFT indicators among males and females respectively. Although the majority of BMR values were lower among those who have low values of LFT indicators, statistical analysis showed insignificant relation (*p* > 0.05).

**Table 1:** Characteristics of males and females in the study group

Parameter	Male n = 98	Female n = 103	P value
Age (year)	19.84 ± 1.30	20.27 ± 1.32	0.020
Height (cm)	173.13 ± 6.04	157.94 ± 5.45	<0.001
Weight (kg)	83.85 ± 22.25	56.55 ± 11.04	<0.001
BMI (kg/m^2^)	27.89 ± 6.99	22.62 ± 3.98	<0.001
FVC (L)	3.65 ± .916	3.06 ± .910	<0.001
FEV1 (L)	3.21 ± 0.72	2.77 ± 0.68	<0.001
FEV1/FVC %	89.09 ± 8.65	92.03 ± 8.18	0.014
FEF25 (L/s)	5.56 ± 2.09	4.82 ± 1.50	0.004
FEF50 (L/s)	4.45 ± 1.22	3.96 ± 0.94	0.002
FEF75 (L/s)	2.36 ± 0.81	2.24 ± 0.70	0.254
FEF25-75 (L/s)	3.90 ± 1.05	3.54 ± 0.87	0.007
FEF75-85(L/s)	1.89 ± 0.81	1.83 ± 0.70	0.592
PEFR (L/s)	6.06 ± 1.97	5.18 ± 1.56	<0.001
Harris-Benedict (kcal/m^2^/h)	41.20 ± 1.67	37.25 ± 0.87	<0.001
Mifflin (kcal/m^2^/h)	38.70 ± 0.42	34.42 ± 0.28	<0.001
FAO/WHO/UN (kcal/m^2^/h)	41.33 ± 2.31	35.36 ± 1.29	<0.001
Henery-Rees (kcal/m^2^/h)	37.79 ± 1.88	37.45 ± 0.84	<0.001

**Table 2:** Comparison between overweight/obese and non-obese subjects among males and females in the study group

Parameter	Group	Male			Female		
		Mean	SD	P value	Mean	SD	P value
Age (year)	Non-obese	19.70	1.424	0.365	20.33	1.308	0.443
	Overweight/obese	19.94	1.188		20.11	1.370	
Height (cm)	Non-obese	172.02	6.090	0.101	157.57	5.566	0.263
	Overweight/obese	174.04	5.895		158.93	5.084	
Weight (kg)	Non-obese	64.14	8.290	<0.001	51.47	6.729	<0.001
	Overweight/obese	99.91	16.265		70.18	8.499	
BMI (kg/m^2^)	Non-obese	21.66	2.411	<0.001	20.70	2.255	<0.001
	Overweight/obese	32.98	5.107		27.76	2.880	
FEV1 (L)	Non-obese	3.22	0.726	0.898	2.73	0.643	0.381
	Overweight/obese	3.20	0.727		2.86	0.769	
Ratio (%)	Non-obese	90.06	9.617	0.321	92.40	8.617	0.452
	Overweight/obese	88.31	7.783		91.03	6.929	
Fef25 (L/s)	Non-obese	5.45	1.846	0.645	4.70	1.471	0.208
	Overweight/obese	5.65	2.274		5.12	1.574	
Fef50 (L/s)	Non-obese	4.51	1.308	0.631	3.88	0.857	0.158
	Overweight/obese	4.39	1.160		4.18	1.126	
Fef75 (L/s)	Non-obese	2.54	0.906	0.043	2.25	0.704	0.804
	Overweight/obese	2.21	0.695		2.21	0.710	
FEF25_75 (L/s)	Non-obese	4.08	1.149	0.140	3.46	0.835	0.166
	Overweight/obese	3.76	0.939		3.73	0.942	
FEF75_85 (L/s)	Non-obese	2.08	0.904	0.033	1.85	0.698	0.599
	Overweight/obese	1.73	0.688		1.77	0.697	
Pefr (L/s)	Non-obese	5.82	1.874	0.272	5.08	1.518	0.281
	Overweight/obese	6.26	2.035		5.45	1.646

**Table 3:** The estimated basal metabolic rate among overweight/obese and non-obese subjects using four different predictive equations

Equation	Group	Male			Female		
		Mean	SD	P value	Mean	SD	P value
Harris-Benedict (kcal/day)	Non-obese	1,676	133	<0.001	1,344	70	<0.001
	Overweight/obese	2,176	234		1,526	86	
Harris-Benedict (kcal/m^2^/h)	Non-obese	40	0.5	<0.001	37	0.9	0.019
	Overweight/obese	42	1.3		37	0.6	
Mifflin (kcal/day)	Non-obese	1,623	108	<0.001	1,237	91	<0.001
	Overweight/obese	1,992	177		1,434	105	
Mifflin (kcal/m^2^/h)	Non-obese	39	0.3	<0.001	34	0.2	<0.001
	Overweight/obese	39	0.5		35	0.3	
FAO/WHO/UNU (kcal/day)	Non-obese	1,659	127	<0.001	1,253	99	<0.001
	Overweight/obese	2,206	249		1,528	125	
FAO/WHO/UNU (kcal/m^2^/h)	Non-obese	39	0.9	<0.001	35	0.8	<0.001
	Overweight/obese	43	1.8		37	1.0	
Henry and Rees (kcal/day)	Non-obese	1,527	111	<0.001	1,202	77	<0.001
	Overweight/obese	2,005	218		1,417	97	
Henry and Rees (kcal/m^2^/h)	Non-obese	36	0.8	<0.001	33	0.8	<0.001
	Overweight/obese	39	1.5		34	0.7

**Table 4:** Predicted basal metabolic rate (BMR) in males in relation to indicators of lung function tests (LFT)

LFT indicator		BMR predictive equation (kcal/m^2^/h)			
		Harris-Benedict	Mifflin	FAO/WHO/UN	Henry-Rees
FVC (L)	Normal (30)	41.52 ± 1.83	38.70 ± 0.52	41.82 ± 2.43	38.17 ± 2.01
	Low (68)	41.06 ± 1.58	38.70 ± 0.37	41.12 ± 2.24	37.62 ± 1.81
	P value	0.215	0.971	0.172	0.183
FEV1 (L)	Normal (35)	41.13 ± 1.72	38.63 ± 0.46	41.14 ± 2.38	37.61 ± 1.96
	Low (63)	41.24 ± 1.65	38.74 ± 0.39	41.44 ± 2.29	37.89 ± 1.84
	P value	0.750	0.224	0.537	0.478
FEF25 (L/s)	Normal (72)	41.22 ± 1.66	38.71 ± 0.44	41.35 ± 2.30	37.80 ± 1.88
	Low (26)	41.15 ± 1.72	38.68 ± 0.36	41.30 ± 2.39	37.77 ± 1.93
	P value	0.846	0.818	0.937	0.946
FEF50 (L/s)	Normal (95)	41.22 ± 1.66	38.70 ± 0.42	41.34 ± 2.32	37.79 ± 1.89
	Low (3)	41.02 ± 1.82	38.67 ± 0.40	41.28 ± 2.36	37.79 ± 1.89
	P value	0.737	0.798	0.939	0.997
FEF75 (L/s)	Normal (95)	41.19 ± 1.64	38.70 ± 0.41	41.32 ± 2.26	37.78 ± 1.84
	Low (3)	41.53 ± 2.74	38.80 ± 0.78	41.87 ± 4.28	38.27 ± 3.59
	P value	0.728	0.681	0.688	0.659

**Table 5:** Predicted basal metabolic rate (BMR) in females in relation to indicators of lung function tests (LFT)

LFT indicator		BMR predictive equation (kcal/m^2^/h)			
		Harris-Benedict	Mifflin	FAO/WHO/UN	Henry-Rees
FVC (L)	Normal (49)	37.36 ± 0.85	34.45 ± 0.26	35.53 ± 1.26	33.77 ± 0.86
	Low (54)	37.15 ± 0.88	34.39 ± 0.30	35.20 ± 1.30	33.51 ± 0.80
	P value	0.209	0.345	0.199	0.119
FEV1 (L)	Normal (61)	37.42 ± 0.83	34.42 ± 0.25	35.38 ± 1.22	33.73 ± 0.82
	Low (42)	37.00 ± 0.87	34.41 ± 0.33	35.32 ± 1.39	33.49 ± 0.85
	P value	0.013	0.878	0.828	0.161
FEF25 (L/s)	Normal (94)	37.26 ± 0.86	34.41 ± 0.25	35.32 ± 1.18	33.61 ± 0.79
	Low (9)	37.13 ± 0.99	34.53 ± 0.48	35.77 ± 2.19	33.80 1.27
	P value	0.673	0.204	0.320	0.526
FEF50 (L/s)	Normal (101)	37.25 ± 0.83	34.42 ± 0.28	35.37 ± 1.29	33.64 ± 0.82
	Low (2)	37.25 ± 2.62	34.20 ± 0.00	34.50 ± 0.85	33.30 ± 1.84
	P value	0.999	0.266	0.344	0.576
FEF75 (L/s)	Normal (101)	37.25 ± 0.87	34.42 ± 0.28	35.35 ± 1.28	33.62 ± 0.83
	Low (2)	37.45 ± 0.35	34.50 ± 0.00	35.80 ± 2.26	33.95 ± 1.48
	P value	0.744	0.684	0.625	0.588

## Discussion

Measurement of basal metabolic rate is needed for nutritional assessment, weight loss planning and care for various medical conditions. Because of the increasing awareness about its importance worldwide, a free metabolic rate calculator, based on Mifflin’s equation, was recently released as a new application for smart mobile phones [15]. Unfortunately, there is a paucity of data regarding measurement of metabolic rate for patients admitted to clinical facilities in Saudi Arabia, and there is no nationally validated predictive equation for obese or non-obese subjects. In this study, we chose four commonly used predictive equations for estimation of BMR in our study. In general, predictive equations tend to overestimate the actual energy expenditure; however, Mifflin equation may be more accurate than the other equations by about 5 % [1].

The finding that lung function indicators are significantly higher among the males compared to the females is a normal physiological finding. Other factors known to affect lung volumes and capacities are age, height and weight. The age range of the students who participated in our study is relatively narrow (from 18 to 23 years old). This decreases variation in their expected values of lung function indicators; however, the range of height is wide. The negative effects of excessive weight gain on the respiratory system are well known [8–10]. Accordingly, we divided our students into overweight/obese and non-obese subjects. In the absence of extreme values, presentation of lung function indicators in the form of (mean ± SD) is representative for our findings in the study population. Our finding that, there was an insignificant difference in lung ventilatory function between overweight/obese and non-obese subjects was similar to results of a study recently conducted in king Abdulaziz medical city in Riyadh, Saudi Arabia [16]. The researcher recommended searching for another diagnosis to explain abnormal lung function findings in obese subjects [16].

Basal metabolic rate is relatively constant but varies greatly between individuals depending on their age, gender, and free fat mass. Gender is a significant determinant of BMR, with men having a greater rate than women [17]. However, this difference might be attributed to higher free fat mass in males compared to females. Since the estimation of BMR in this study was based on predictive equations, results were significantly higher among males compared to females. Higher BMR values among males were reported by Arciero and his colleagues in 1993 who conducted their study in the United States across a broad spectrum of age [18]. In Arciero study, the lower values in females persisted even after controlling for differences in body composition and aerobic fitness.

It is well known that patients who consume low amount of oxygen have lower rate of metabolism than those who consume higher amounts; however, patients with impaired lung function, have still higher BMR compared to those with normal lung function, due to the increased activity of their respiratory muscles as a compensation for the impaired lung function. A recent study has shown that patients with COPD and bronchial asthma have increased metabolic rate that is directly correlated to severity of the diseases [19]. On the other hand, the negative effect of abnormal metabolism on lung function was confirmed in many previous studies. For example, diabetic patients had FEV1 values lower than those without diabetes, and this effect is even greater in those with poorly controlled diabetes [20]. In contradistinction to these results, we found insignificant association between predicted BMR and abnormal values of lung function indicators. However, a significant difference in predicted BMR between overweight/obese and non-obese subjects is an expected finding since BMR calculations are directly based on weights of the participants. It is worth noting that accurate BMR calculations should rely on free fat mass rather than total body weight; that is why many studies found that the predictive equations overestimate the BMR, especially in obese subjects [21].

## Conclusion

In conclusion, this study showed that the relation between predicted basal metabolic rate and indicators of lung function tests was statistically insignificant. A practical estimation of BMR based on direct measurement of oxygen consumption is recommended to confirm the absence of this association.
